# Investigating the Efficacy and Safety of Oral Cicaglocal on Wound Healing After Mohs Surgery in Patients With Skin Cancer: A Randomized, Double‐Blinded, Placebo‐Controlled Clinical Trial

**DOI:** 10.1111/jocd.16784

**Published:** 2025-02-07

**Authors:** Ali Asilian, Parisa Mohammadian, Hadiseh Mahram, Reza Shahriarirad, Mohammad Bigham

**Affiliations:** ^1^ Department of Dermatology, School of Medicine Isfahan University of Medical Sciences Isfahan Iran; ^2^ Department of Dermatology, Skin and Leishmaniasis Research Center Isfahan University of Medical Sciences Isfahan Iran; ^3^ School of Medicine Shiraz University of Medical Sciences Shiraz Iran; ^4^ Thoracic and Vascular Surgery Research Center Shiraz University of Medical Sciences Shiraz Iran; ^5^ Biotechnology Research Center Shiraz University of Medical Sciences Shiraz Iran

**Keywords:** bromelain, Centella, hyaluronic acid, Mohs surgery, wound healing

## Abstract

**Background:**

Mohs micrographic surgery is the gold standard treatment for skin cancers in cosmetically sensitive anatomic areas, with a cure rate close to 100%. Managing post‐Mohs surgery wounds can pose challenges, particularly in elderly patients. This study evaluates the Cicaglocal drug, a supplement with bromelain, *Centella asiatica* , hyaluronan, vitamins, and minerals, for enhancing wound healing post‐Mohs surgery.

**Method:**

This double‐blinded, randomized clinical trial conducted at Al‐Zahra Hospital in 2023 involved 24 patients aged 50–80 with SCC or BCC undergoing Mohs surgery. Patients were randomized into two groups Cicaglocal and placebo. Treatments were administered postoperatively, and outcomes such as erythema reduction, early healing score (EHS), full recovery, and patient and physician satisfaction levels were evaluated 7 and 14 days after initiation. Data were collected through clinical assessments and standardized wound photographs.

**Result:**

Oral Cicaglocal demonstrated significantly improved outcomes compared to their respective placebo groups in terms of erythema score, EHS, full recovery score, and patient and physician satisfaction levels.

**Conclusion:**

Cicaglocal can enhance wound healing and lead to improved clinical outcomes following Mohs micrographic surgery.

## 1 Introduction

Mohs micrographic surgery is a tissue‐sparing surgical method for managing skin cancers, including basal cell carcinomas (BCC) and squamous cell carcinomas (SCC) [1, 2]. The procedure involves precise microscopic mapping along with staged removal, resulting in the highest cure rate (close to 100%) while optimizing the preservation of normal, uninvolved tissue [3]. Despite the benefits of Mohs surgery, postoperative wound healing can be challenging due to factors such as tumor size, location, depth, patient age, and comorbidities [4]. Therefore, developing effective strategies to enhance patient outcomes following Mohs surgery is crucial.

Wound healing is accomplished through a complex, well‐regulated cascade, including hemostasis/coagulation, inflammatory, proliferative, and remodeling phases [5]. The process is initiated almost immediately after the surgery [6]. However, in some cases, the wound fails to progress through a regular sequence and often gets stuck in the inflammatory phase, ultimately resulting in the development of a chronic wound [5]. Chronic wounds, taking longer than 12 weeks to heal, are usually associated with several risk factors, with age and diabetes being the primary ones [7]. They are a significant public health concern associated with high economic and psychosocial burdens [8]. Thus, appropriate treatment is essential to improve wound healing and prevent further complications.

The Cicaglocal drug is a supplement formulated with various components, including bromelain as its main active ingredient, *Centella asiatica* (Asiaticosides), sodium hyaluronan, ascorbic acid, B vitamins (B2, B3, B7), cholecalciferol, and minerals such as iron, zinc, and copper. Bromelain is a major sulfhydryl proteolytic enzyme extracted from the stem of the pineapple plant ( *Ananas comosus* ) consisting of a complex mixture of different thiol‐endopeptidases, phosphatases, glucosidases, peroxidases, cellulases, glycoproteins, and carbohydrates [9]. Furthermore, it has demonstrated the potential to enhance wound‐healing activity through the efficient debridement of necrotic tissue and the modulation of inflammatory reactions [10]. Another component of the Cicaglocal drug is *Centella asiatica* extracts, which have been shown to positively impact wound healing by improving collagen synthesis and microcirculatory function [11].

Hyaluronan, also called sodium hyaluronate, is a major extracellular matrix macromolecule and accelerates wound healing by promoting cell migration and proliferation, enhancing granulation tissue formation, and reducing inflammation [12]. Moreover, Cicaglocal also contains vitamins and minerals crucial for cellular repair and immune function in wound healing [13]. These components create a synergistic effect that enhances the body's natural wound‐healing mechanisms, contributing to the overall efficacy of the Cicaglocal drug.

In this clinical trial, we aim to evaluate the effect of oral Cicaglocal drugs in patients after Mohs surgery. This study seeks to contribute valuable insights into wound management strategies, ultimately enhancing postsurgical care for patients with skin cancer.

## 2 Method and Patients

### 2.1 Study Design and Participants

This study was conducted as a double‐blinded, randomized clinical trial. Patients were recruited from the dermatology outpatient clinic at Al‐Zahra Hospital, affiliated with Isfahan University of Medical Sciences, during a 3‐month period in 2023 (October 2023—December 2023). We selected our participants among patients aged 50–80 with dermatological conditions of the head and face in which a diagnosis of skin cancer (SCC or BCC) was established, and they were scheduled to undergo Mohs surgery at the operation room of Al‐Zahra Hospital. Patients with certain underlying diseases that directly impair wound healing, such as scleroderma and morphea, were excluded from the study. We also excluded patients with skin cancers other than SCC and BCC and those who had Mohs surgery on body parts other than the head and face. In addition, patients with treatment‐related complications or unwillingness to continue the study were withdrawn from the study. The study was approved by the ethics committee of Isfahan University of Medical Sciences (Ethical code: IR.ARI.MUI.REC.1402.291) and also registered on the Iranian Registry of Clinical Trial database (Code: IRCT20220306054206N3; Registered on 2024‐02‐26; available at: https://irct.behdasht.gov.ir/trial/75349). All patients who agreed to participate in the study were required to sign a written informed consent form. Based on a review of previous studies on individual components of this drug, it is noted that this combination of Cicaglocal for wound healing has not been independently tested in a clinical trial. Therefore, this pilot study was conducted on two groups of 12 patients each.

### 2.2 Randomization and Blinding

Patients were allocated into two study groups, each consisting of 12 individuals, using simple randomization via the Random Allocation software. Patients were randomly selected by lottery corresponding to either the oral Cicaglocal group or the oral placebo group. The study was conducted in a double‐blind manner, in which neither the researcher, who administered the medication, nor the patient knew whether the active drug or placebo was being administered.

Cicaglocal and placebo treatments were produced in identical capsule, with the placebo medicine prepared using the same container as the original medicine, with only its contents being replaced with a placebo. Starch was employed to formulate oral medication in capsule format.

### 2.3 Interventions

Treatment was initiated on the first postoperative day. Patients were instructed to consume their designated capsules once daily and were encouraged to do so simultaneously every day to maintain consistency. Patients continued their usual medications, with the study drug added. Cicaglocal was manufactured by Daru Darman Parshin Pod Company and contains bromelain as its main ingredient, along with vitamin C, B‐group vitamins (B2, B3, B7), vitamin D, iron, zinc, copper, Centella plant extract, and sodium hyaluronate. Patients were provided with detailed instructions on how to take the medication properly during each session, and the usage method was also indicated on the product. During the study, all patients were advised to inform the researchers immediately in case of any adverse effects, such as irritation or erythema. In such cases, treatment would be promptly discontinued, and the patient would receive standard medical care until recovery, ensuring their safety and well‐being.

### 2.4 Data Collection

Eligible patients were evaluated regarding their demographic characteristics (age, gender, occupation) and clinical information (type of cancer leading to surgery, presence of diabetes and hypertension). In addition, posttreatment information, including wound healing index, full recovery, erythema score, and patient and physician satisfaction, was collected using a checklist completed by the collaborating physician at each follow‐up.

Patients underwent preintervention consultations as well as follow‐up assessments at 7 and 14 days after the initiation of treatment. Standardized wound photographs were taken from a distance of 20 cm during these visits. A collaborating physician then would conduct a thorough clinical assessment, evaluating the wound healing index, full recovery, erythema score, patient and physician satisfaction, and identifying any potential side effects. The evaluation of wound healing employed the early healing score (EHS) [14, 15], which is based on the assessment of clinical signs of re‐epithelialization, hemostasis, and inflammation, encompassed a rating scale from 1 (indicative of worst possible healing) to 10 (indicative of ideal healing). Additional parameters, including erythema, full recovery, and patient and physician satisfaction levels, were also assessed on a scale of 1–10.

### 2.5 Data Analysis

The collected data were entered into SPSS software version 25 for analysis. Quantitative data were reported as mean ± standard deviation and qualitative data were reported as *N* (%). The chi‐square test or Fisher's exact test was utilized to compare qualitative variables, while a one‐way ANOVA was used to compare quantitative variables. A one‐way repeated measures ANOVA test was also used to compare continuous variables at different time points. A *p*‐value of less than 0.05 was considered statistically significant.

## 3 Results

### 3.1 Patient Allocation and Grouping

**FIGURE 1:**
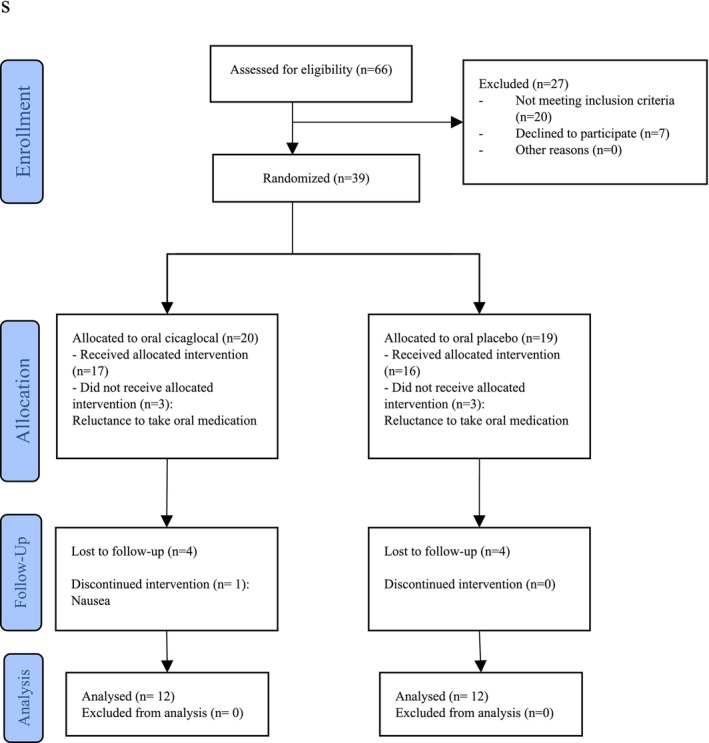
Study flow diagram of patients undergoing Mohs surgery due to squamous cell and basal cell carcinoma.

In the present study, a total of 24 patients who underwent Mohs surgery were assessed for eligibility. Subsequently, we randomly assigned 12 individuals to either the treatment oral Cicaglocal or placebo group. The CONSORT chart in Figure 1 represents the allocation of patients in our study.

### 3.2 Demographical and Clinical Features

Ultimately, 24 patients, including 11 women (45.8%) and 13 men (54.2) with a mean age of 67.0 ± 8.1 years (range: 50–80 years), were included in the analysis. No statistically significant difference was observed between the two groups regarding their gender, age, cancer type, occupation, and the presence of diabetes and hypertension. This suggests that randomization successfully balanced the distribution of patients across groups. Demographic and baseline characteristics of patients are summarized in Table 1.

**TABLE 1:** Demographic and clinical characteristics of patients undergoing Mohs surgery due to squamous cell and basal cell carcinoma.

Variable	Total; *N = 48*	Treatment groups		
		Oral cicaglocal	Oral placebo	*p* ^a^
Age (year); mean ± SD	67.4 ± 6.9	66.0 ± 7.8	67.9 ± 8.5	0.57
Gender; *n* (%)				
Female	11 (45.8)	4 (33.3)	7 (58.3)	0.22
Male	13 (54.2)	8 (66.7)	5 (41.7)	
Occupation; *n* (%)				
Business	4 (16.7)	1 (8.3)	3 (25.0)	0.34
Farmer	1 (4.2)	0 (0)	1 (8.3)	
Housewife	11 (45.8)	4 (33.3)	7 (58.3)	
Retired	8 (33.3)	5 (41.7)	3 (25.0)	
Cancer type; *n* (%)				
BCC	19 (79.2)	9 (75.0)	10 (83.3)	1.00
SCC	5 (20.8)	3 (25.0)	2 (16.7)	
Diabetes mellitus; *n* (%)	8 (33.3)	4 (33.3)	4 (33.3)	1.00
Hypertension; *n* (%)	14 (58.3)	8 (66.7)	6 (50.0)	0.41

Abbreviations: BCC, basal cell carcinoma; SCC, squamous cell carcinoma; SD, standard deviation.

^a^ *p*‐Values are calculated based on Chi‐square/Fisher's exact test or independent sample *t*‐test.

### 3.3 Outcome Evaluation

Only one patient reported adverse side effects in our study, a case of nausea in the oral Cicaglocal group, which caused the cessation and change of mediation. There was no significant association between the side effects and the groups in our study (*p* = 1.000).

**FIGURE 2:**
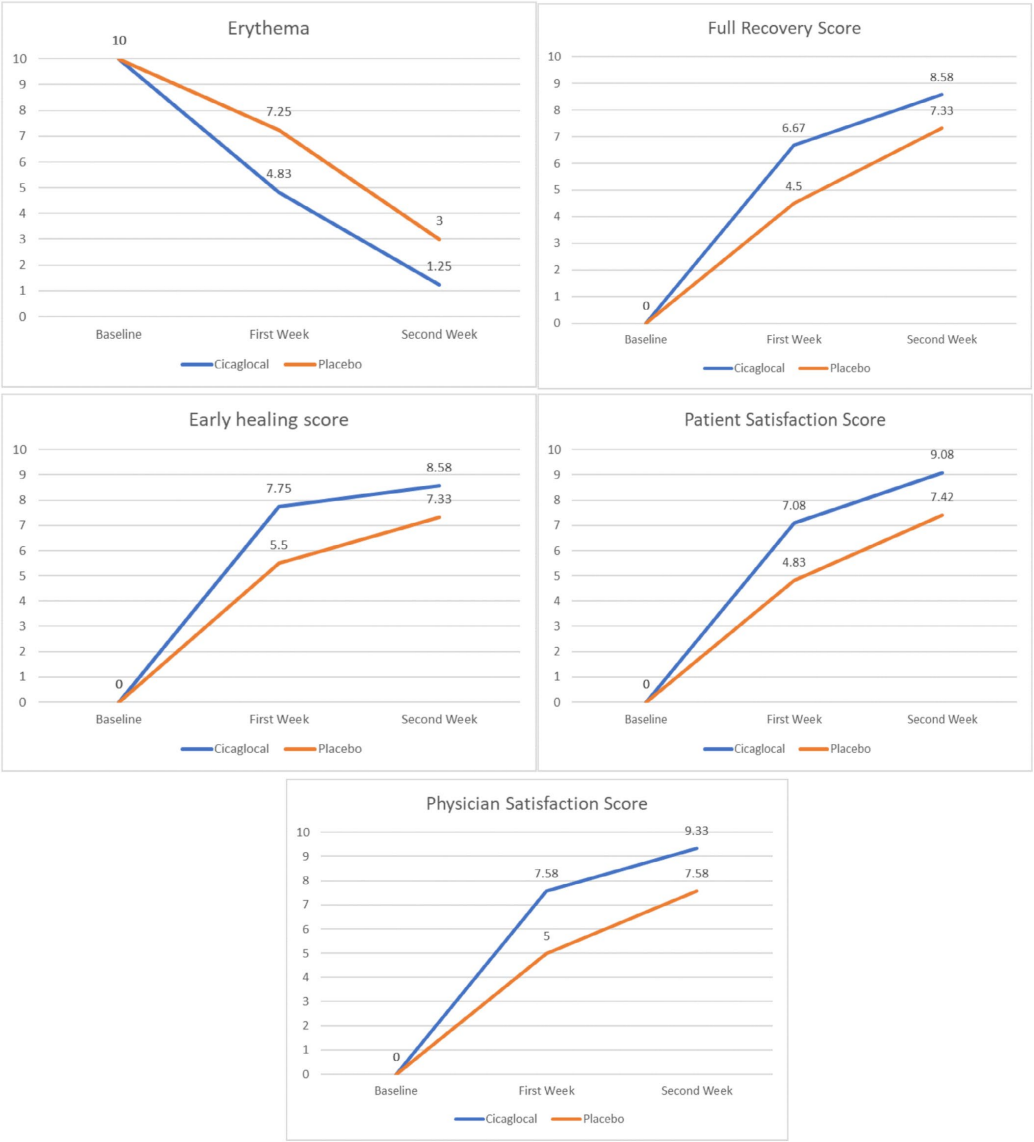
Comparison of the average of erythema score, early healing score, full recovery score, physician satisfaction score, and patient satisfaction score changes between study groups during the study period based on a general linear model.

Erythema scores significantly decreased at each time point in all treatment groups (*p* < 0.001), and the amount of change differed significantly among the groups (*p* < 0.001). Figure 2 demonstrates the erythema changes during the study period. The mean erythema score in the oral Cicaglocal group decreased from 4.83 after 1 week to 1.25 after 2 weeks. The Cicaglocal group showed significantly less erythema compared to the placebo group (*p* < 0.001).

As shown in Figure 2, EHS significantly increased from the start of the study to the second and final visits in the treatment group (*p* < 0.001). The changes in EHS also varied significantly across the groups (*p* < 0.001). The mean EHS score improved from 7.75 after 1 week to 8.58 after 2 weeks in the oral Cicaglocal group, with significantly better EHS than the placebo groups (*p* < 0.001).

Regarding achieving full recovery, the results revealed a significant increase in full recovery scores at both the second and final visits (*p* < 0.001) (Figure 2). The study groups also exhibited significant variations in the level of full recovery score change (*p* < 0.001). The mean full recovery scores improved from 6.67 on the second visit to 8.58 on the last visit in the oral Cicaglocal group, with significantly better full recovery scores than the placebo groups (*p* < 0.001).

During the study period, there was a significant increase in both physician and patient satisfaction levels (*p* < 0.001) (Figure 2). When comparing physician satisfaction levels between the groups during the treatment period, the oral Cicaglocal groups reported significantly higher scores at the second and final visits compared to the placebo group (*p* < 0.001). The results for patient satisfaction levels were similar, with the oral Cicaglocal groups demonstrating significantly higher scores at the second and final visits compared to the placebo group (*p* < 0.001).

**FIGURE 3:**
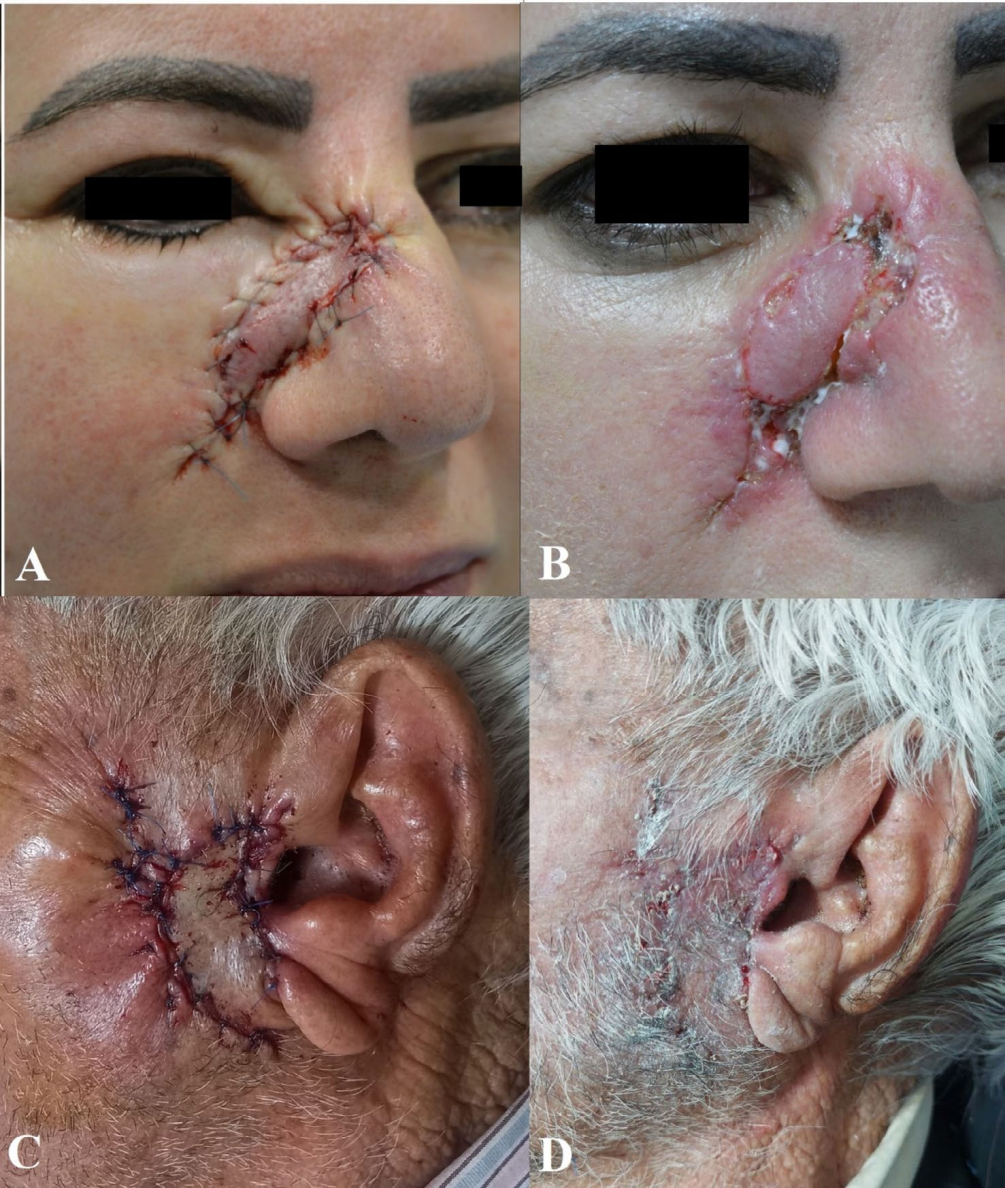
Comparison of the effects of Cicaglocal and placebo on wound healing after Mohs surgery. Left side images (A and C) show the postoperation state, while the right images show wound healing after 2 weeks with placebo (B) and Cicaglocal (D).

Figure 3 illustrates two patients from our study receiving either Cicaglocal or a placebo. As demonstrated, the improvement in the Cicaglocal group is more satisfactory compared to the placebo group.

## 4 Discussion

Despite the many advantages of Mohs surgery, it can lead to complications such as scarring and delayed wound healing, particularly in sensitive areas such as the face, neck, and chest [16]. Careful decision‐making is essential to choose the proper treatment for post‐Mohs micrographic surgery wounds. To address this issue, we evaluated the effect of oral Cicaglocal drugs on wound healing in patients after Mohs surgery. The results demonstrated that oral Cicaglocal groups exhibited significantly improved outcomes compared to the placebo group regarding erythema reduction, enhanced EHS, and increased full recovery scores.

Bromelain, a key component of the Cicaglocal drug, exhibits various therapeutic effects, such as anti‐inflammatory, fibrinolytic, and debridement activities [9]. Its anti‐inflammatory effect is closely linked to its ability to reduce bradykinin levels at inflammation sites and decrease prekallikrein levels in serum [9]. In a clinical trial on patients undergoing cataract surgery, oral administration of bromelain 2 days before surgery and 5 days postoperatively resulted in significant inflammation and pain reduction [17]. Additionally, a clinical trial investigating the effect of bromelain on wound healing after episiotomy demonstrated that wound healing was faster in the treatment group than in the placebo group [18]. Consistent with previous studies, the observed reduction of erythema and accelerated full recovery can be attributed to the effect of bromelain in the Cicaglocal drug.

Additionally, *Centella Asiatica* extract in the formulation may have contributed to erythema reduction due to its anti‐inflammatory effect. Asiatic acid is one of its main chemical components and plays a pivotal role in its wound‐healing activity. Experimental studies have shown that administering *Centella Asiatica* extract topically or orally can effectively reduce neutrophil recruitment, lower the release of TNF‐α, IL‐1β, IL‐6, and IgE, and inhibit the expression of iNOS, COX‐2, NF‐κB, and lipoxygenase (LOX) activity at the site of skin injury [19]. In a randomized clinical trial on the effect of *Centella Asiatica* extract postlaser resurfacing wound healing on the face, the treatment group exhibited significantly less erythema index [20]. In line with these studies, our findings underscore the potential impact of this component in decreasing erythema after Mohs surgery.

*Centella asiatica* extract is also proven to be involved in the proliferative and remodeling phases of wound healing through collagen synthesis, simulation of extracellular matrix accumulation, and maintenance of granulation tissue [11]. A randomized clinical trial revealed that a polymeric spray film solution containing *Centella Asiatica* extract was advantageous for acute wound treatment, accelerating healing time without adverse effects [21]. In the present study, the significant improvement in EHS and full recovery scores in the Cicaglocal groups indicates that, aside from the anti‐inflammatory effect of *Centella asiatica* extract, it also contributed to promoting the overall wound‐healing process.

Hyaluronan, another component of the study drug, is believed to play a role in each stage of wound healing. Several studies have shown that topical administration of hyaluronic acid significantly improves wound healing. For instance, in patients with second‐degree burns, applying hyaluronic acid in addition to silver sulfadiazine cream resulted in a significantly shorter healing time than silver sulfadiazine alone [22]. Similarly, hyaluronic acid dressings in venous ulcers showed a faster and more significant reduction in ulcer dimensions compared with a standard wound dressing (dextranomer paste) [23]. Hyaluronic acid has also been demonstrated to be effective in its oral supplement form [24]. These findings highlight the efficacy of hyaluronic acid in enhancing wound healing and support its inclusion in the Cicaglocal drug formulation.

The effect of the Cicaglocal components, including bromelain, *Centella asiatica* , and hyaluronic acid, on wound healing has been studied individually. In our research, we focused on exploring the impact of the combination of these components on wound healing. Our findings revealed a significant improvement in the overall healing process. This positive outcome can be attributed to the synergistic action of these components, which have individually shown the ability to promote cell proliferation, migration, and the deposition of extracellular matrix. Moreover, the presence of vitamins and minerals in the Cicaglocal formulation, such as vitamin C, vitamin D, and zinc, may have further contributed to this beneficial effect by supporting cellular metabolism, collagen synthesis, and immune function, all of which are crucial for optimal wound healing [13].

Our study limitation includes the small sample size, and lack of molecular or dermoscopic evaluation. However, our study was a pilot study for the introduction of Cicaglocal in the field of Mohs surgery and wound care, and can be employed as the cornerstone for further larger population studies.

## 5 Conclusion

Our study demonstrated that oral administration of Cicaglocal drug can improve different aspects of wound healing, including reduced erythema and inflammation, enhanced epithelialization, and overall accelerated recovery after Mohs surgery. Moreover, the high satisfaction rates reported by both patients and physicians, in conjunction with the favorable safety profile of the Cicaglocal drug, suggest its potential as a valuable adjunct therapy for post‐Mohs surgery patients. However, a larger sample size is needed to assess and confirm these findings comprehensively.

## Ethics Statement

The research ethics committee of Isfahan University of Medical Sciences has approved this study (Ethical code: IR.ARI.MUI.REC.1402.291) and it was also registered on the Iranian Registry of Clinical Trial database (Code: IRCT20220306054206N3; Registered on 2024‐02‐26; available at: https://irct.behdasht.gov.ir/trial/75349).

## Consent

The authors have nothing to repot.

## Conflicts of Interest

The authors declare no conflicts of interest.

## Data Availability

The data supporting this study's findings are available from the corresponding author upon reasonable request.
